# Zebrafish (*Danio rerio*) embryo-larvae locomotor activity data analysis: Evaluating anxiolytic effects of the antidepressant compound citalopram

**DOI:** 10.1016/j.dib.2019.104812

**Published:** 2019-11-15

**Authors:** Johannes Pohl

**Affiliations:** Department of Biomedical Sciences and Veterinary Public Health, Swedish University of Agricultural Sciences, Uppsala, Sweden

**Keywords:** Zebrafish, Behavior, Locomotor activity, Developmental biology, Developmental neurotoxicology

## Abstract

Newly fertilized zebrafish (*Danio rerio*) embryos were exposed to increasing concentrations of the selective serotonin reuptake inhibitor (SSRI) citalopram from fertilization until six days post-fertilization (dpf). Locomotor activity data were acquired at six dpf using an automated ZebraBox® infrared tracking system. Individual (n = 32) locomotor activity was recorded during 75 minutes in total during alternating illumination conditions (0% light, i.e. dark periods, and 100% light, i.e. light periods). The first 15 minutes of the test consisted of a dark period, i.e an acclimatization phase. Afterward, six alternating light and dark periods were conducted. Individual zebrafish embryo-larvae locomotion was tracked and aggregated in ten-second bins. The dataset, containing nine locomotor-related quantified endpoints (factors), was parsed, analyzed, and visualized using R software. The dataset and its associated custom R script may be used to further explore locomotor activity outcomes (e.g. anxiolytic or anxiogenic properties) following exposure to citalopram or other neuroactive chemicals.

**Table:** Specifications Table

Subject	Biology
Specific subject area	Behavioral Neuroscience, Developmental Neuroscience, Developmental Neurotoxicology, Developmental Toxicology, Aquatic toxicology, Environmental Toxicology, Pharmacology, Toxicology
Type of data	Figure
How data were acquired	Infrared tracking using ZebraBox® apparatus with Zebralab software (Viewpoint, Lyon, France)
Data format	Comma-separated value (.csv) data files
Parameters for data collection	Zebrafish embryo-larvae displaying obvious malformations (e.g. non-inflated swim bladder) or other visible effects (e.g. mortality) were omitted from data analysis
Description of data collection	Fertilized zebrafish embryos were placed individually in 96-well plates together with 200 μL test solution. After six days static exposure, the plates were carefully transferred to the ZebraBox® testing chamber. Individual embryo-larvae locomotor activity was tracked during six alternating ten-minute dark and light periods. Raw data files (.csv) were exported after tracking. Data analysis was performed using a custom R script
Data source location	Institution: Swedish University of Agricultural Sciences City/Town/Region: Uppsala Country: Sweden
Data accessibility	Repository name: Mendeley Data Data identification number: 10.17632/rcscvwcyx4.2 Direct URL to data: https://data.mendeley.com/datasets/rcscvwcyx4/2
Related research article	Bachour, R.-L., Golovko, O., Kellner, M., Pohl, J., 2020. Behavioral effects of citalopram, tramadol, and binary mixture in zebrafish (*Danio rerio*) larvae. Chemosphere 238. https://doi.org/10.1016/j.chemosphere.2019.124587

**Table:** 

**Value of the Data** • Fish embryo locomotor activity is a sensitive measurement of anxiogenic and/or anxiolytic properties of chemical compounds, in the present case citalopram. • The data and associated R scripts are made available to researchers who would like to reproduce analysis of high-throughput fish embryo-larvae locomotor activity datasets. • The supplied raw-data files may be further analyzed to extract additional endpoints, further elucidating neurobehavioral outcomes of citalopram exposure during embryonal development.

## 1 Data

**Fig. 1:**
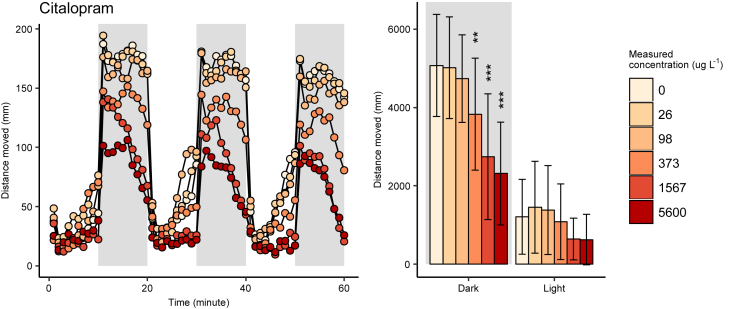
Total swimming distance in six-day post-fertilization zebrafish embryo-larvae exposed to citalopram since fertilization (**: p<0.01, ***: p<0.001 according to Dunnett's post hoc test). Adapted from Bachour et al. (2020) [2].

The raw comma-separated value (.csv) data files associated with this article (citalopram_plateA.csv and citalopram_plateB.csv) are deposited in Mendeley Data [1]. An R script (behavior_analysis.R) for data analysis of the .csv files is also available from Mendeley Data [1]. The plotted data showing locomotor activity in zebrafish embryo-larvae exposed to citalopram is shown in Fig. 1.

## 2 Experimental design, materials, and methods

### 2.1 Zebrafish embryo procurement

Groups of adult zebrafish (*Danio rerio*) from the laboratory breeding stocks were transferred to metal mesh (5 mm mesh size) breeding cages submerged in 10 L aquariums (26 °C) on the day before each test. Spawning was induced among the groups of fish (approximately five males and five females) after lights-on the following morning. After spawning, fertilization success and malformation rate was assessed in each spawning group, and the group displaying the lowest incidence of unfit eggs were collected and processed for embryo exposure. The eggs were rinsed in fresh carbon-filtered aerated tap water to remove debris from the spawning aquariums. Exposure in test solutions began at approximately 3 hours post-fertilization when the embryos had reached the 1k-cell to “high” developmental stage [3]. Embryos showing synchronous development were sorted out for testing. The embryo exposure tests were performed within the window approved by the local animal welfare authority, i.e. before the external feeding developmental phase.

### 2.2 Embryo exposure

**Fig. 2:**
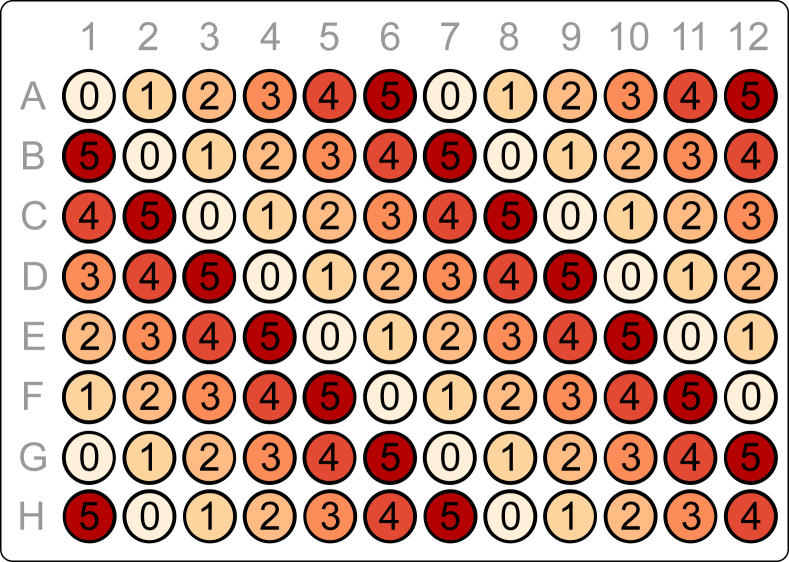
Placement of embryos in each 96-well plate, where each well is denoted by treatment group ranging from control (0) to the highest tested chemical concentration (5).

Fertilized embryos (*n* = 32 per treatment) were distributed in Petri-dishes filled with 40 mL test solution (control and five chemical treatments). Control water consisted of carbon-filtered aerated tap water (pH: 8.36±0.04, conductivity: 450±30 mS/cm, alkalinity: 8 °dH, dissolved O_2_: 90±5%). Citalopram was dissolved in control water by mixing and vortex, without the addition of an additional solvent, and diluted 1:4 into the five treatments. Embryos were pipetted from each respective petri dish together with 200 μL solution into 96-well plates (one embryo per well). The water column in each well reached 2/3 of the well height (well volume: 300 μL) allowing for an air column, thus facilitating normal swim bladder inflation in the developing embryo-larvae [4]. The treatment groups were organized in diagonal rows in order to even out positional bias (e.g. differences in temperature in the center of the plate compared with the edge of the plate) as illustrated in Fig. 2. 16 individual replicates for each treatment were placed on each of two 96-well plates.

The plates were covered in Parafilm M (Bemis Company, United States) and kept in an environmentally controlled laboratory (12:12 h daylight cycle (09:00–21:00), 26±1 °C air temperature). The plates were incubated until the embryo-larvae reached six days post-fertilization (dpf) when the locomotor activity assay was performed.

### 2.3 Locomotor activity assay

**Fig. 3:**
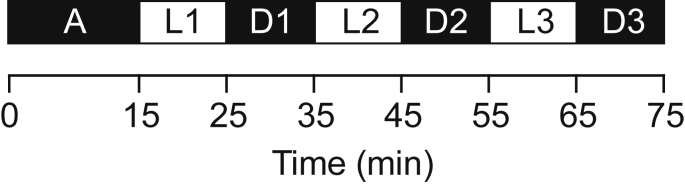
Timeline of the locomotor activity assay protocol consisting of an acclimatization step (A), and six alternating light (L1-L3) and dark (D1-D3) periods.

The locomotor activity assays were conducted at six dpf. The plates were handled minimally before placement in the ZebraBox® recording chamber (ViewPoint Life Sciences, Lyon, France). The recording chamber was equipped with an infrared light-emitting floor and a top-mounted infrared camera, which enabled a consistent video recording of the whole plate under both light and dark conditions. The ZebraLab software (Viewpoint Life Sciences, Lyon, France) was used to program the light cycle protocol, consisting of a 15 minute initial acclimatization step, and six alternating ten-minute light and dark phases (Fig. 3).

Zebrafish embryo-larvae will react to the instantaneous shift from 100% to 0% illumination by an increase in locomotor activity. Anxiolytic compounds (e.g. citalopram) can reduce this response causing a decreased activity during dark periods as compared to control. Anxiogenic compounds may, on the other hand, further induce hyperactivity in embryo-larvae during dark periods [5].

The assays were performed in the early afternoon (12:00–15:00) as embryo-larvae tend to respond to illumination changes with the least variance during that time of day [6]. Three activity thresholds were defined for locomotion; inactivity (movement speed <3 mm/s), small movements (movement speed 3–6 mm), and large movements (movement speed >6 mm/s). Zebrafish embryo-larvae locomotor data were aggregated in ten-second bins for each individual, separated into nine locomotor relevant activity variables (Table 1). Following the completion of the 75-minute assay, the 96-well plates were removed from the recording chamber. A high dose of pH buffered Tricaine mesylate (MS-222) was added into each well incapacitating the embryo-larvae, before morphological assessment by stereo microscopy.

**Table 1:** Locomotor relevant activity variables in the.csv raw data files [1].

Variable abbreviation	Variable definition	Unit
inact	Inactivity bouts (<3 mm/s)	count
inadur	Inactivity duration (<3 mm/s)	s
inadist	Inactivity distance (<3 mm/s)	mm
smlct	Small movement bouts (3–6 mm/s)	count
smldur	Small movement duration (3–6 mm/s)	s
smldist	Small movement distance (3–6 mm/s)	mm
Larct	Large movement bouts (>6 mm/s)	count
lardur	Large movement duration (>6 mm/s)	s
lardist	Large movement distance (>6 mm/s)	mm

### 2.4 Locomotor activity data processing

Raw.csv data files were exported following the locomotor activity assays [1]. All data filtering and analysis were performed using R software [7] with the RStudio interface [8] and published R packages (Table 2). Detailed data processing procedures are contained within the supplied R script behavior_analysis.R [1].

**Table 2:** R packages used for data analysis and visualization.

Package name	Functionality	URL	Reference
Base R	Fundamental programming tools	https://r-project.org	[7]
ggplot2	Data visualization	https://ggplot2.tidyverse.org	[9]
multcomp	Statistical analysis, i.e. Dunnett's post-hoc test	http://multcomp.r-forge.r-project.org	[10]
plyr	Splitting and combining data frames	http://had.co.nz/plyr/	[11]

**Fig. 4:**
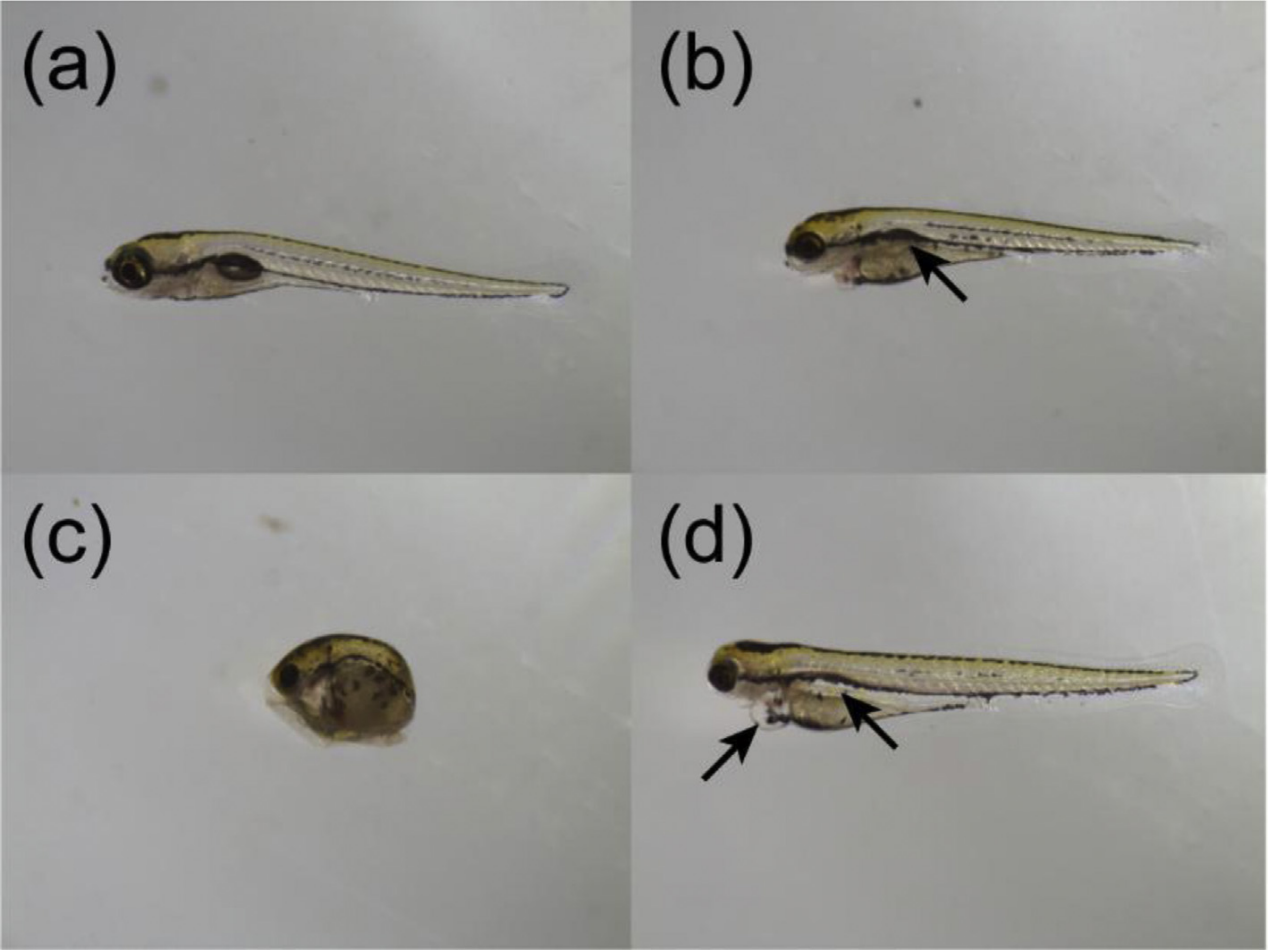
Representative morphological images of (a) normal 6 dpf embryo-larvae, (b) lack of swim-bladder inflation, (c) coagulated/dead, and (d) pericardial- and yolk sac edemas.

In short, the .csv files were imported, aggregated, and combined. Observations from morphologically affected individuals (e.g. swim-bladder not inflated) were omitted from the datasets in order not to confound the data analysis (Fig. 4). Individual total swimming distance (defined as smldist + lardist) during each minute and light and dark periods were summarized. Control and treatment mean±standard deviation were then plotted using the ggplot2 package [9].

### 2.5 Statistical analysis

Locomotor activity data (i.e. total swimming distance during dark conditions) was summarized for each individual embryo-larvae replicate and analyzed using one-way analysis of variance (ANOVA) followed by normal distribution evaluation. Significant treatment effects were assessed by Dunnett's post hoc test using the multcomp R package [10]. Treatment groups with p-values of <0.05 (*), <0.01 (**), and <0.001 (***) were considered as significantly deviating from the control mean.
